# A Mini Review: Application Progress of Magnetic Graphene Three-Dimensional Materials for Water Purification

**DOI:** 10.3389/fchem.2020.595643

**Published:** 2020-11-19

**Authors:** Biao Wang, Qingwang Liu, Zhenzhong Fan

**Affiliations:** Petroleum Engineering College, Northeast Petroleum University, Daqing, China

**Keywords:** graphene, magnetic, water purification, aerogel, sponge

## Abstract

Marine oil pollution, colored counterattacks, and heavy metal ions in the water will cause serious environmental problems and threaten human health. The three-dimensional material prepared by graphene, as a new nanomaterial, has a large specific surface area and surface chemical activity. Various impurities in the water can be absorbed, which is very suitable as a water purification material. Depositing Fe_3_O_4_ and other magnetic materials on graphene three-dimensional materials can not only increase recyclability but increase hydrophobicity. Therefore, magnetic graphene three-dimensional materials have a high potential for use in water purification. This article reviews the research progress and adsorption mechanism of magnetic graphene materials for water purification. Finally, the future research prospects of magnetic graphene materials have prospected.

## Introduction

In recent years, the increasing human activities have posed a huge threat to the ecological environment. Among the rest, water pollution is the most common problem in the world (Chen et al., 2018). The oil (Fahid et al., 2020), dyes (Noreen et al., 2020), and heavy metal ions (Lgarashi et al., 2020) are the three main factors causing the water pollution. Offshore oil drilling (Guo et al., 2020) and oil spills in marine transportation (Lubetkin, 2020) are serious threats to the marine ecosystem. The development of the textile industry (Afshari and Dinari, 2020), mining engineering (Collins et al., 2020), and machinery industry (Xie et al., 2020) has also brought a large amount of dye waste liquid and heavy metal ion wastewater that are difficult to handle, which brings huge dangers to the ecological environment and human health. In all the applications of the graphene gels and sponges, high performance in adsorption capacity for oil, heavy metals, and synthetic dyes from water has particularly aroused much concern.

The traditional water purification methods mainly include ozone (Kim et al., 2020), membrane filtration (Zhao et al., 2020), flocculation (Song et al., 2020), biological adsorption (Martin et al., 2020) and electrochemical methods (Chen et al., 2020). Although these water purification technologies have many advantages, they have the problems of high cost, short life, poor reusability, and harsh reaction conditions. Besides, these technologies are not efficient in treating large amounts of wastewater. Among mentioned methods, adsorption process is one of the most preferred methods which can be used for many pollutant uptake. Graphene is a two-dimensional hydrophobic and lipophilic material (Granato et al., 2016), it has a large specific surface area that making them excellent adsorbent material (Song et al., 2012). In the current experiment, graphene three-dimensional materials have high removal efficiency of crude oil, metal ions and dyes in water (Xiaolin et al., 2009; Bong et al., 2015; Xing et al., 2016; Gu et al., 2019), which can be considered as a novel high adsorption capacity material of up to 90–100% (Alammar et al., 2020). The adsorption material can be removed by extrusion and distillation to realize the recycling of the adsorbent and the adsorbed material.

Zhao et al. (2017) prepared a graphene microsphere that can be mass-produced using a wet spinning method. It can not only absorb 70–195 times of its weight of oil or other organic dyes, but can be reused after 1,000 extrusion cycles. This kind of aerogel with a unique core structure has excellent adsorption and reusability performance. Harvesting graphene sponges and gels by magnetic separation regard as a promising approach because of the rapid, easy and efficient capture of adsorption material from liquid solution applied by an external magnetic field. Therefore, how to increase the magnetic properties of graphene three-dimensional materials to achieve recycling is a hot research topic in water purification. This article will review the application of graphene-made magnetic aerogels and sponges in water purification, summarize the current research status, this will have a guiding significance in the field of water purification.

## Graphene Three-Dimensional Materials: Graphene Aerogels and Graphene Sponges

Graphene aerogels and sponges are three-dimensional materials with large specific surface areas (Chen et al., 2018) and unique porous structure (Xiao et al., 2017). The multiple actions of capillary driving force, π-π bonds, hydrogen bonds, van der Waals forces and electrostatic forces cause the materials play various important roles for water remediation applications (Guo et al., 2016).

Many studies have shown that the capillary driving force (Homaeigohar and Elbahri, 2017) in the nanochannels of graphene materials makes the transfer rate of water molecules between graphene sheets 4–5 orders of magnitude diffusion (Kannam et al., 2012; O'Hern et al., 2012; Sun et al., 2015). This consequently leads to the good hydrophobic properties of the material. Due to a abundant of oxygen-containing functional groups in graphene oxide (GO) (Han et al., 2013), water molecules are likely to form hydrogen bonds with the oxygen-containing functional groups on the GO sheet to adsorb water molecules. Therefore, the hydrophobic properties of three-dimensional materials are associated the degree of reduction of oxygen-containing functional groups in GO (Wang et al., 2018).

Oil field wastewater usually contains a large amount of asphaltenes (Groenzin and Mullins, 1999), forming a solid oil-water interface film. Graphene can absorb asphaltenes in the oil through the interaction of π-π bonds and electrostatic forces (Wang et al., 2016), thereby breaking the oil-water interface film and adsorbing asphaltenes, dirt oil and other impurities. The interaction between the π-π bond and the electrostatic force is also the key force for the adsorption of colored dyes on water (Yang et al., 2018).

Also, since graphene aerogels and sponges have a large number of active sites (Cai et al., 2008), these sites provide a way for the adsorption of metal ions and colored dyes (Han et al., 2018). Meanwhile, the carboxyl groups on the graphene surface layer are negatively charged due to ionization (Huang et al., 2015), working with hydroxyl groups can absorb pollutants in water through the action of hydrogen bonds (Wu et al., 2017) and electrostatic force (Tabrizi and Zamani, 2016).

The theoretical specific surface area of single-layer graphene oxide is 2,630 m^2^/g (Shu et al., 2017), so the graphene-based three-dimensional material has more oxygen-containing functional groups, extremely higher porosity, unique porous structure, larger specific surface area and more adsorption sites than traditional adsorption materials. These combinations make graphene an excellent adsorption material to adsorb a variety of macromolecular organics in water. The current methods for preparing graphene aerogels and sponges include chemical precipitation, hydrothermal, chemical grafting, oil phase decomposition, and dip coating, etc. (Zhang et al., 2020).

## Magnetic Graphene Aerogel

The oil, harmful metal ions and dyes contaminants in river and ocean threaten ecological environment and human health. How to remove these harmful substances in water and reduce environmental pollution is a hot issue in the field of environmental protection. Compared with the single adsorption mechanism of traditional adsorbents, graphene aerogels and impurities in water have the following forces: electrostatic interaction, physical adsorption, ion exchange, chemical coordination, hydrogen bonds, π-π bonds and van der Waals forces. These forces are coordinated with each other, one force is the main force, and the other is assisted, which can absorb most of the dirty oil, organic solvents, dyes or various metal ions in the water.

Slop oil in water is a macromolecular substance, and the quantity of oil is huge. If adsorbents are used to adsorb oil in water, solvothermal method for preparing graphene aerogel (GA) is an excellent choice. The reaction conditions of the solvothermal method are relatively simple, various silane coupling agents can be added in the reaction to graft hydrophobic chains in the graphene gel to increase the hydrophobic properties of the adsorbent. Zhou et al. (2015) reported a GA/ Fe_3_O_4_/Polystyrene Composites with a density of 0.005 g/cm^−3^ (99.7% volume porosity) using a solvothermal method. This aerogel has a good adsorption and recycling performance on diesel oil, lubricating oil and crude oil in water. It also has a magnetic and a better hydrophobic performance than aerogels without Fe_3_O_4_, making the aerogels have better adsorption performance and can be absorbed by magnets and recycled.

For metal ions in water, the adsorbent needs to have a large number of adsorption sites. As a magnetic graphene aerogel with many adsorption sites, it still has good adsorption performance. Simple solvothermal method cannot greatly increase the adsorption sites of GA, so soapless emulsion polymerization and modified massart method is better for increasing adsorption sites. Pan et al. (2018) used soapless emulsion polymerization to synthesize an as-prepared calcium alginate/GO composite aerogel. Due to the ion exchange and chemical coordination, this aerogel has extremely high adsorption performance for Pb^2+^, Cu^2+^, and Cd^2+^in water. The removal rates of the three ions in this water are 95.4, 81.2, and 73.2% respectively, among the top ranks of the current adsorbents. Seema (2020) prepared a magnetic prussian blue/reduced graphene oxide (3D-MPBRGO) with a specific surface area of 402.68 m^2^/g by modified massart method. The adsorption capacity of CS^+^ on the magnetic graphene aerogel obtained by self-assembly is as high as 484.12 mg/g.

**Figure 1:**
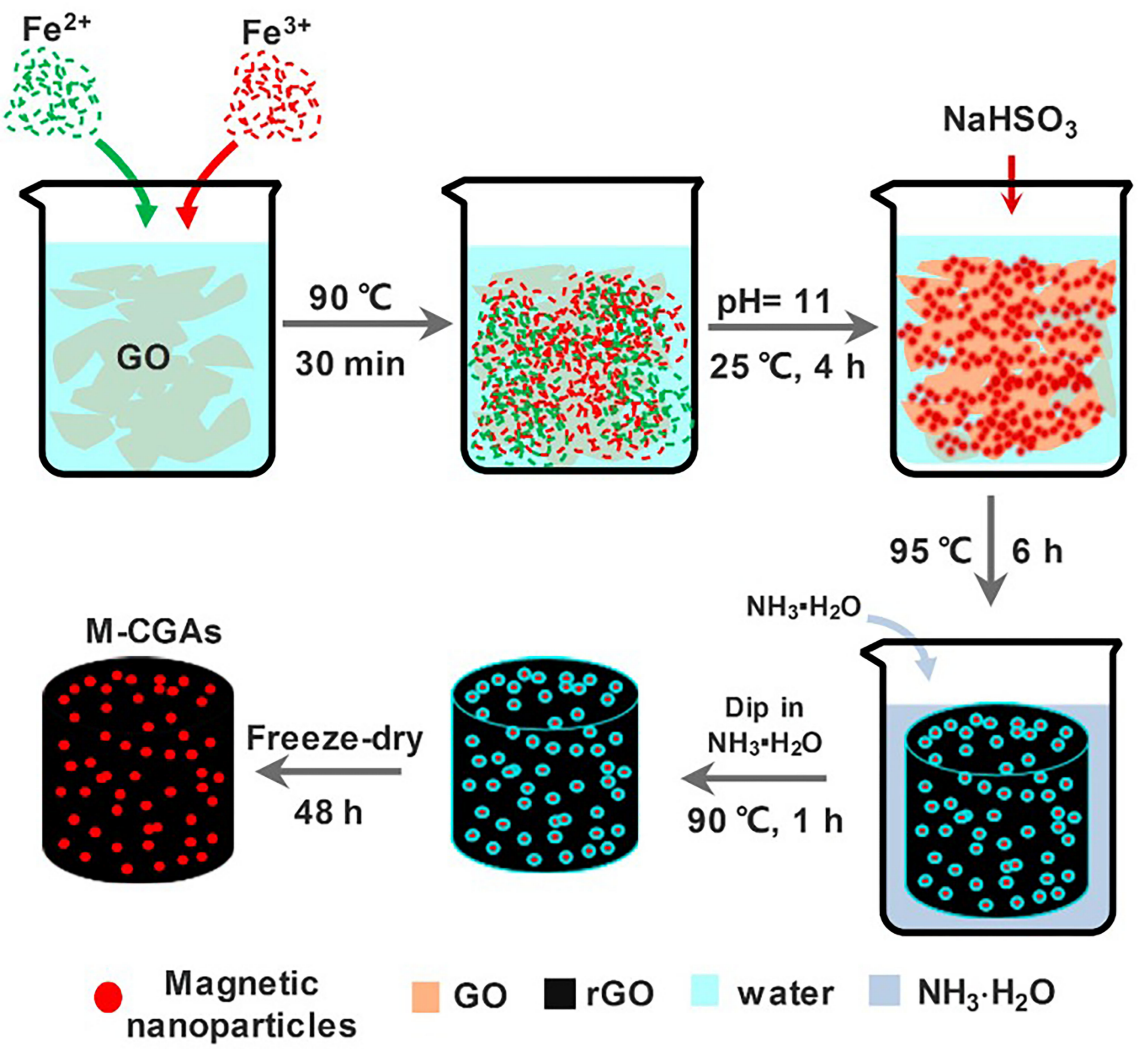
Schematic illustration demonstrating the synthesis process of magnetic composite graphene aerogels (M-CGAs) (Liu et al., 2019).

Since graphene has good hydrogen bonds and π-π bonds between dyes, graphene gel also has good adsorption properties for colored dyes. Arabkhani and Asfaram (2020) synthesized a magnetic graphene aerogel with Fe(NO_3_)_3_ and bacterial cellulose nanofiber through the facile filler-loaded networks method with the vacuum freeze-drying method, which can remove 93% malachite green in water. It still has 62.7% adsorption capacity after 7 cycles, with extremely high adsorption performance and recyclability. Liu et al. (2019) used hydrothermal-coreduction method, a simple and environmentally friendly method, to prepare a gel for adsorption of methylene blue, within 180 min, Figure 1 is schematic illustration demonstrating the synthesis process of magnetic composite graphene aerogels. Besides, Xiong et al. (2020) obtained a magnetic aerogel with opening-channels exposed with amphiprotic active groups through Ionically Mediate Self-assembly. This type of gel can not only adsorb Congo red /Methylene blue but also Cu^2+^, Pb^2+^, Cd^2+^, Cr^3+^, and other metal ions.

At present, the hydrothermal reduction method is used to prepare graphene gel, its preparation method is simple, obtained product has good adsorption performance for macromolecular organic matter. But the adsorption effect for small molecule impurities is not obvious. Therefore, it is necessary to adopt methods such as soapless emulsion polymerization, facile filler-loaded networks method with the vacuum freeze-drying to increase the adsorption sites of graphene, thereby increasing the various effects of graphene on metal ions and colored dyes.

## Magnetic Graphene Sponge

Compared with graphene gels, graphene sponges mostly use polyurethane sponges and metal organic frameworks as substrates. It is synthesized using Immersion Method, coprecipitation, or lyophilization methods, which rely on electrostatic interaction and physical adsorption combined to adsorb impurities. Although graphene sponge is difficult to separate oil and water from emulsified oil, its performance in adsorbing oil, metal ions and colored dyes is better than graphene aerogel. the doping of graphene can increase the strength and anti-pollution resistance of the sponge (Carreño et al., 2015; Yu et al., 2015, 2017), too.

The immersion method is the most common in the preparation of graphene sponge. At a certain temperature, graphene oxide will agglomerate and can coagulate onto the sponge skeleton. When we choose some sponges with hydroxyl groups, simultaneous esterification of carboxyl and hydroxyl groups with GA and polyurethane sponge will also proceed at the same time. This series of reactions allows graphene oxide to be firmly grafted onto the polyurethane sponge by the immersion method. Anju and Renuka, 2020 connected anchoring graphene-meso iron oxide and polyurethane sponge through a simple immersion method, obtained a magnetic graphene sponge with excellent recyclability, which can be recycled up to an astonishing 150 times. Liu et al. (2015) show that graphene sponge prepared by Immersion method can absorb lubricating oil, paraffin oil, hydraulic oil, peanut oil, N-hexadecane, hexane, octane, heptane and other oils and organic solvents. In the future, graphene sponge can play a good role in daily cleaning and handling of crude oil spills accidents.

Although the adsorption of metal ions and colored dyes by sponges is not as good as aerogels with a large number of adsorption sites, due to the low cost of sponges, there are still scholars trying to increase adsorption sites on the sponges, expecting to obtain cheaper ions and dye adsorbent. Sarkar et al. (2019) doped functionalized chitosan and poly (methacrylic acid) into graphene, which greatly improved the adsorption performance of methylene blue. The adsorption capacity of sponge is 2.478 g/g, which is currently the best adsorbent for removing cationic dyes from water. This experiment verified that the sponge can also adsorb colored dyes by increasing the adsorption sites, the adsorption performance is also excellent.

With the increasing scarcity of energy, studies have found that oil and other sources of energy can be extracted from a large number of oil-bearing algae (Seo et al., 2015). Therefore, looking for adsorbents that adsorb oil algae in the ocean is also a hot topic in energy extraction. Liu et al. (2016) used the high speed mixing method to combine cationic polymer PDDA (diallyldimethylammonium chloride) and Fe_3_O_4_ grafted into graphene sponge. A magnetic graphene sponge with excellent adsorption capacity for oily seaweed is obtained. It can still maintain good adsorption performance after 5 times usage.

Sponges have good adsorption properties for all liquids. Therefore, we usually use dip coating or co-precipitation methods to graft graphene particles onto the sponge skeleton, to obtain a sponge with good hydrophobicity and lipophilicity. The adsorption performance of sponge to macromolecular organics is much greater than that of gel. It is of great importance to develop a sponge with better ability to adsorb metal ions and other small molecule impurities, realizing more economical sponges with merged merits of hydrothermal reduction, free radical polymerization approach and other methods. Graphene is expensive, but the cost of preparing graphene sponge is not very high. Although the adsorption performance is not very amazing, its excellent recyclability determines its prospects. I believe that graphene sponge can be used as an excellent adsorbent into industrial production in the next decade.

## Conclusion and Outlook

Nowadays, graphene is modified by chemical cross-linking or the addition of metal oxide nanoparticles to obtain better adsorption performance. In Table 1, there is summary of different magnetic graphene three-dimensional material and their properties. In the treatment of marine oil pollution and ecological environment water treatment, graphene gel and sponge have significant adsorption ability on oil pollution, organic solvents, cationic dyes and other pollutants. Grafting Fe_3_O_4_ or various nano-ferrites on the one hand increases the roughness but also increases the magnetic properties on the gel and sponge, and increases the recyclability of the material in water. In general, magnetic graphene three-dimensional materials will be one of the main directions of future water purification research.

**Table 1:** Summary of different magnetic graphene three-dimensional material and their properties (Carreño et al., 2015; Liu et al., 2015, 2016, 2019; Ye et al., 2015; Yu et al., 2015, 2017; Zhou et al., 2015; Pan et al., 2018; Cai et al., 2019; Sarkar et al., 2019; Anju and Renuka, 2020; Arabkhani and Asfaram, 2020; Seema, 2020; Xiong et al., 2020).

**Name**	**Materials**	**Preparation methods**	**Separation of the material**	**Adsorption mechanism**	**Adsorption capacity (g/g)**	**Separation capacities (times)**
GA/Fe_3_O_4_/ polystyrene composites	FeCl_3_·6H_2_O	Solvothermal technique	Oil	Electrostatic interactions/physical adsorption	30–47	10
As-prepared calcium alginate/GO composite aerogel	Sodium alginate	Soapless emulsion polymerization	Pb^2+^/Cu^2+^/Cd^2+^	Ion exchange/CCE	0.37/0.10/ 0.19	20
Magnetic bacterial cellulose nanofiber/ GO polymer aerogel	Fe(NO_3_)_3_/bacterial cellulose nanofiber	Facile filler-loaded networks method with the vacuum freeze-drying	Malachite green	H-bonding/ π-π interactions, electrostatic interactions/physical adsorption	0.27	7
Magnetic composite GAs	FeCl_2_·4H_2_O/FeCl_3_·6 H_2_O	Hydrothermal-coreduction method	MB	π-π interactions/H-bonding	0.09	/
3D-MPBRGO	Iron chloride/ethanol potassium ferricyanide	Modified massart method	Cs^+^	Chemical adsorption	0.48	/
Fe_3_O_4_/GA	FeC_2_O_4_·2H_2_O	Hydrothermal method	Arsenic	Physical adsorption	0.04	/
Magnetic aerogel	Amphiprotic microcrystalline cellulose/Fe_3_O_4_	Ionically mediate self-assembly	Congo red/MB/Cu^2+^/Pb^2+^/ Cd^2+^/Cr^3+^	H-bonding/vander waals interactions/physical adsorption	0.28/0.35/ 0.22/ 0.57/ 0.19/0.12	5
GO-Fe_3_O_4_/ PDDA	FeCl_2_·4H_2_O/FeCl_3_·6 H_2_O	High speed mixing method	Algae	Electrostatic interactions	14.06	5
GIOPF	Fe_3_O_4_/poly urethane sponge	Immersion method	Diesel oil/ chloroform	Physical adsorption.	90–316	150
SOGMS	1H,1H,2H,2H-P/ poly urethane sponge	Immersion method	Oil	Physical adsorption.	80.80	20
Magnetic polymer-based graphene foam	FeSO_4_·7H_2_O/ octadecyltrichl orosilane	Hydrothermal reduction/immersion method	Paraffin oil/peanut oil/hexane/octane/ heptane	Physical adsorption.	9–27	8
Fe–rGO sponge	Dimethylsiloxane/ metallic Fe–graphite core–shell nanoparticles/ascorbic acid	Hydrothermal reduction/immersion method	RB	H-bonding/ Van Der Waals/π-π interactions/ electrostatic interactions	0.01	/
Fe_3_O_4_-GS	FeCl_2_·4H_2_O/ FeCl_3_·6H_2_O	Lyophilization	MB	Electrostatic interactions	0.53	10
Cl–CS–p(MA)/ Fe_3_O_4_NPs	Chitosan/methacrylic acid/FeCl_3_·6H_2_O/ FeSO_4_·7H_2_O	Free radical polymerization approach/hydrothermal reduction	MB	Electrostatic interactions	2.48	/
MGOS	Fe_3_O_4_	Coprecipitation/ lyophilization	Tetracycline	Physical adsorption/ π-π interaction/ H-bonding/electrostatic interactions	0.47	/

*CCE, Chemical Coordination Effects; MB, Methylene Blue; RB, Rhodamine B; GIOPF, Graphene-meso Iron Oxide composite incorporated Polyurethane Foam; SOGMS, Super hydrophilic/Oleophobic Graphene-based Magnetic Sponge; 1H,1H,2H,2H-P, 1H,1H,2H,2H-Perfluorooctyltrimethoxysilane; MGOS, Magnetic Graphene Oxide Sponge*.

(1) Studies have shown that the adsorption effect of graphene three-dimensional materials is affected by pH and temperature, and its performance is better in alkaline environments. It is susceptible to a certain degree of corrosion in acidic media and interferes with adsorption performance. Therefore, the graphene three-dimensional material can be doped with magnetic SiO_2_ nanomaterials that can protect the material in an acid environment, at the same time increase the active sites and magnetic properties, and improve the adsorption performance and recyclability.

(2) In the aspect of adding magnetism to graphene, Fe^2+^ and Fe^3+^ are often used to generate Fe_3_O_4_. Studies have shown that the hydroxylation of metal ions in ferrite can selectively adsorb different metal ions and cationic dyes is better. Considering the adsorption of metal ions and cationic dyes, ferrites such as MnFe_2_O_4_, NiFe_2_O_4_, CoFe_2_O_4_ can be used instead of Fe_3_O_4_.

(3) In the purification of oil fields, industrial water, and domestic sewage, ordinary graphene three-dimensional materials are susceptible to bacterial corrosion. It is a good suggestion to add silver to graphene three-dimensional materials to increase the sterilization ability of graphene.

(4) At present, oil spills on the ocean occur frequently, magnetic graphene sponge is a good way to deal with marine oil spills due to its simple preparation method, excellent adsorption performance, easy recovery of external magnetic field and excellent recycling performance.

(5) The graphene three-dimensional material itself has a large specific surface area and a large number of adsorption sites. Furthermore, it has the effect of electrostatic force, hydrogen bond, van der Waals force, π-π Interactions and other forces on various impurities in water. At present, research on its adsorption mechanism believes that these forces are mainly one force, and other forces play an auxiliary role. The combined effect of these forces has a good adsorption effect on impurities in water, but the mechanism is not very in-depth and further works are required.

## Author Contributions

BW contributed to the conception of the study, contributed significantly to analysis, manuscript preparation, performed the data analyses, and wrote the manuscript. QL and ZF helped perform the analysis with constructive discussions. All authors contributed to the article and approved the submitted version.

## Conflict of Interest

The authors declare that the research was conducted in the absence of any commercial or financial relationships that could be construed as a potential conflict of interest.
